# Influence of childhood maltreatment on prevalence, onset, and persistence of psychiatric comorbidities and suicide attempts in bipolar disorders

**DOI:** 10.1192/j.eurpsy.2022.7

**Published:** 2022-01-22

**Authors:** D. Grillault Laroche, O. Godin, Y. Dansou, R. Belzeaux, B. Aouizerate, T. Burté, P. Courtet, C. Dubertret, E. Haffen, P. M. Llorca, E. Olie, P. Roux, M. Polosan, R. Schwan, M. Leboyer, F. Bellivier, C. Marie-Claire, B. Etain, The FACE-BD Collaborators

**Affiliations:** 1 AP-HP, Groupe Hospitalo-Universitaire AP-HP Nord, DMU Neurosciences, Hôpital Fernand Widal, Département de Psychiatrie et de Médecine Addictologique, Paris, France; 2 Université de Paris, INSERM UMR-S 1144, Optimisation Thérapeutique en Neuropsychopharmacologie OTeN, Paris, France; 3 Univ Paris Est Créteil, INSERM U955, IMRB, Translational NeuroPsychiatry Laboratory, Créteil, France; 4 AP-HP, Hôpitaux Universitaires Henri Mondor, Département Médico-Universitaire de Psychiatrie et d’Addictologie (DMU IMPACT), Fédération Hospitalo-Universitaire de Médecine de Précision en Psychiatrie (FHU ADAPT), Créteil, France; 5 Fondation FondaMental, Créteil, France; 6 Pôle de Psychiatrie, Assistance Publique Hôpitaux de Marseille, Marseille, France; 7 INT-UMR7289, CNRS Aix-Marseille Université, Marseille, France; 8 Centre Hospitalier Charles Perrens, Bordeaux, France; 9 Centre Hospitalier Charles Perrens, Laboratoire NutriNeuro (UMR INRA 1286), Université de Bordeaux, Bordeaux, France; 10 Pôle de Psychiatrie, Centre Hospitalier Princesse Grace, Monaco, France; 11 Department of Emergency Psychiatry and Acute Care, CHU Montpellier, IGF, Univ. Montpellier, CNRS, INSERM, Montpellier, France; 12 Université de Paris, Paris, France; 13 AP-HP, Groupe Hospitalo-Universitaire AP-HP Nord, DMU ESPRIT, Service de Psychiatrie et Addictologie, Hôpital Louis Mourier, Colombes, France; 14 Université de Paris, Inserm UMR1266, Sorbonne Paris Cité, Faculté de Médecine, Paris, France; 15 Service de Psychiatrie de l’Adulte, CIC-1431 INSERM, CHU de Besançon, Laboratoire de Neurosciences, UFC, UBFC, Besançon, France; 16 Centre Hospitalier et Universitaire, Département de Psychiatrie, Clermont-Ferrand, France; 17 Université d’Auvergne, EA 7280, Clermont-Ferrand, France; 18 Centre Hospitalier de Versailles, Service Universitaire de Psychiatrie d’Adulte et d’Addictologie, Le Chesnay, France; 19 Equipe DisAP-PsyDev, CESP, Université Versailles Saint-Quentin-en-Yvelines - Paris-Saclay, Inserm, Villejuif, France; 20 Univ. Grenoble Alpes, Inserm, U1216, CHU Grenoble Alpes, Grenoble Institut Neurosciences, Grenoble, France; 21 Université de Lorraine, Centre Psychothérapique de Nancy, Inserm U1254, Nancy, France

**Keywords:** bipolar disorder, childhood maltreatment, comorbidities, prevalence, suicide

## Abstract

**Background:**

Psychiatric comorbidities and suicide attempts are highly prevalent in Bipolar Disorders (BD). We examined the associations between childhood maltreatment, psychiatric comorbidities, and suicide attempts, in terms of lifetime prevalence, sequence of onset, and current symptoms.

**Methods:**

We assessed 3,047 individuals with BD for suicide attempts, anxiety disorders, substance use disorders, and eating disorders. Participants completed a self-report for the assessment of childhood maltreatment. Associations between childhood maltreatment and characteristics of comorbidities (lifetime prevalence, current symptoms, and age at onset) were examined using logistic regressions and network analyses.

**Results:**

Psychiatric comorbidities were frequent with a mean number per individual of 1.23 (SD = 1.4). Most comorbidities occurred prior to the onset of BD. Participants who reported higher levels of childhood maltreatment had more frequent and multiple comorbidities, which were also more currently active at inclusion. Childhood maltreatment did not decrease the age of onset of comorbidities, but was associated with a faster accumulation of comorbidities prior to the onset of BD. Logistic regression and network analyses showed that emotional abuse and sexual abuse might play a prominent role in the lifetime prevalence of psychiatric comorbidities and suicide attempts.

**Conclusions:**

Childhood maltreatment was associated with suicide attempts, and with frequent, multiple, and persistent psychiatric comorbidities that accumulated more rapidly prior to the onset of BD. Hence, childhood maltreatment should be systematically assessed in individuals with BD, in particular when the course of the disorder is characterized by a high comorbid profile or by a high suicidality.

## Introduction

Bipolar disorders (BD) are very frequently associated with other psychiatric conditions, including anxiety disorders, substance use disorders, and eating disorders, and also frequently associated with suicidal attempts, which is not considered as a “comorbidity” *per se*, but rather an indicator of the severity of the illness [1,2]. Several meta-analyses have reported associations between BD and comorbid psychiatric conditions in large pooled samples. For instance, Alcohol, Cannabis, and/or Substance Use Disorders (AUD, CUD, and SUD) are highly prevalent in individuals with BD, with pooled estimates around 42% for AUD, 20% for CUD, and 17% for SUD [3–5]. The lifetime prevalence of anxiety disorders (of any type) in BD is also very high (being estimated around 40%) and this prevalence remains high even when pooled estimates calculation is restricted to euthymic cases [6–8]. Furthermore, a prevalence of suicide attempts of up to 50% has been reported in BD [9–11].

While prevalence for all psychiatric comorbidities and suicide attempts are well-described in BD, the chronology of onset is poorly known. We have recently reported that most psychiatric comorbidities occurred prior to the onset of BD, with the exception of panic disorders and alcohol use disorders that mainly occurred after the onset of BD [12]. This means that, in BD, most psychiatric comorbidities occurred early in life, i.e. during childhood or adolescence (given a mean age at onset of BD around 25 years old). Determinants that may explain both the high prevalence and the early onset of psychiatric comorbidities in BD remained to be identified. One major putative determinant of these (in terms of both lifetime prevalence and sequence of onset) is childhood maltreatment (also referred as childhood trauma) that is frequently reported by individuals with BD (between 30 and 50% of individuals reported at least one type of abuse) [13–15].

In a sample of individuals with BD that has been collected in France and Norway, we have shown that individuals with comorbid substance use disorders and anxiety disorders had significantly higher CTQ total scores (Childhood Trauma Questionnaire [16]), as compared to individuals without such comorbidities [17, 18]. Furthermore, it has been reported that, among all anxiety disorders, panic disorders were frequently associated with childhood maltreatment in individuals with BD [19]. This association between childhood maltreatment and psychiatric comorbidities has been further confirmed in the meta-analysis published by Agnew-Blais and Danese [20]: when exposed to childhood maltreatment, individuals with BD had more frequent psychiatric comorbidities including alcohol use disorder (OR = 1.44 [1.13–1.83]), SUD (OR = 1.84 [1.41–2.39]), anxiety disorders (OR = 1.90 [1.39–2.61]), and PTSD (post-traumatic stress disorder) (OR = 3.60 [2.45–5.30]), but also more frequent suicidal attempts (OR = 2.26 [1.88–2.70]).

However, several cautions should be considered when interpreting the findings of this meta-analysis. First, various forms of childhood maltreatment (physical abuse, sexual abuse, emotional abuse, neglect, or family conflict/violence) have been pooled into a single category (labeled as childhood maltreatment or childhood adversities). In addition, most comorbidities have also been pooled into one single clinical entity (for instance, any lifetime anxiety disorder or any lifetime substance use disorder). Therefore, there is a need to better clarify the association between fine-grained defined subtypes of childhood maltreatment and fine-grained defined psychiatric comorbidities. Since both subtypes of maltreatment and psychiatric comorbidities were not exclusive, such a disentanglement of the nature of these associations is required. Finally, no meta-analysis explored how childhood maltreatment might modify the sequence of onset of psychiatric comorbidities, given the hypothesis that childhood maltreatment would lead to an earlier onset of psychiatric comorbidities.

The aims of this study were therefore to describe: (a) the associations between childhood abuse and neglect and psychiatric comorbidities (including anxiety, substance use, and eating disorders) and suicide attempts, in terms of lifetime prevalence, sequence of onset, and current level of symptoms, (b) any preferential associations between childhood maltreatment subtypes and lifetime prevalence of psychiatric comorbidities and suicide attempts, and (c) to model the links between childhood maltreatment subtypes and lifetime prevalence of psychiatric comorbidities using network analyses in a large sample of more than 3,000 individuals with bipolar disorders.

## Material and Methods

### Participants

Individuals were recruited in 12 centers of expertise for BD where they have been followed in a cohort called FACE-BD (Fondamental Advanced Centers of Expertise for Bipolar Disorders). All centers used the same systematic and standardized clinical assessments. All individuals were remitted outpatients who were aged 16 years or older and diagnosed with BD (all bipolar types [I, II, and not otherwise specified]) according to DSM-IV criteria [21]. This cohort has been described in details in previous articles [22–24]. Clinical remission was defined by the absence of current hospitalization and the absence of any treatment modification during a period of 8 weeks before inclusion (but not by the absence of any current mood symptoms). The assessment protocol was approved by the institutional review board (Comité de Protection des Personnes Ile de France IX; January 18, 2010), in accordance with the French laws for noninterventional studies and requires only an information letter.

At inclusion, a specialized team (psychiatrists and psychologists) interviewed the individuals using the SCID (Structured Clinical Interview for DSM-IV Axis I Disorders) [25] and systematically recorded information related to the onset and course of BD. Current mood state (depressive and hypomanic symptoms) at inclusion was assessed respectively with the Montgomery Asberg Depression Rating Scale [26] and the Young Mania Rating Scale [27].

The lifetime presence of psychiatric comorbidities and suicide attempts was screened according to the corresponding sections of the SCID. The following disorders were assessed: anxiety disorders (specific phobia, social phobia, agoraphobia, obsessive–compulsive disorder [OCD], generalized anxiety disorder [GAD], and panic disorder), eating disorders (anorexia nervosa, binge eating, and bulimia, then pooled into a single category), post-traumatic stress disorder (PTSD), substance use disorders (alcohol use disorder and cannabis use disorder), and suicide attempt. Each comorbidity was recorded in the database, in terms of (a) lifetime presence according to DSM-IV criteria (presence vs. absence), (b) age at onset (AAO) which was defined as the age at which an individual met for the first time the DSM-IV criteria for a given comorbidity, and (c) current activity (i.e., presence of clinically significant symptoms of each comorbidity in the last month before inclusion).

### Measures of childhood maltreatment

Childhood maltreatment was assessed using the Childhood Trauma Questionnaire (CTQ) [16]. The CTQ is a 28-item self-report that yields a total score (ranging from 25 to 125). Five subscores can be calculated corresponding to emotional abuse, emotional neglect, physical abuse, physical neglect, and sexual abuse. Each subscore can be used as a continuous variable (ranging from 5 to 25) or as a categorical variable defined by four levels of severity (absence, low, moderate, and severe) according to cut-off values. We used the validated French version of the CTQ [28].

### Statistical analyses

The CTQ total score was divided into quartiles with the 75th percentile corresponding to individuals who had experienced the greatest exposure to childhood maltreatment (i.e., multiple and/or severe forms of childhood maltreatment). This use of the CTQ total score divided into quartiles has been previously proposed by References [29 and 30]. Each comorbidity is described in terms of lifetime prevalence (present or absent), age at onset (reported as median and IQR), and current status (not active or active, i.e., presence of clinically significant symptoms during the last month before inclusion). The number of comorbidities per individual was defined as the sum of lifetime comorbidities (excluding suicide attempt).

The associations between quartiles of CTQ total score, prevalence and current activity of psychiatric comorbidities, and suicide attempts were tested using Chi-square or Fisher exact tests. Wilcoxon Mann–Whitney rank tests were used to compare the median age at onset of each comorbidity across quartiles of CTQ total score.

We used multivariable logistic regressions to test for the associations between lifetime prevalence of each comorbidity and the five CTQ subscores (used as categories: absence/low vs. moderate/severe) adjusted for age, gender, BD type, and depressive symptoms at inclusion (MADRS score). Results of associations between comorbidities and CTQ subscores were presented as a heatmap.

Finally, we used a network analysis to model the links between maltreatment subtypes and lifetime psychiatric comorbidities. To harmonize the dataset, we used a “+1 versus −1” binary coding system (for presence vs. absence of a given comorbidity) as recommended for dichotomous variables [31]. We used a network approach for binary data, corresponding to an estimation technique (called eLASSO; least about shrinkage and selection operator) that is based on the IsingFit procedure. The model examines variables that can have two states (+1 and −1 here), with the final model selection based on the extended Bayesian Information Criterion (eBIC). We generated networks diagrams using the Fruchterman and Reingold algorithm. Centrality analysis was computed to test if a given comorbidity or a given subscore CTQ has many and/or strong associations to other comorbidities and/or other CTQ subscores. These variables are more central within the network as compared to a less connected variable. The estimated indices were: Betweenness (the number of times that a node lies on the shortest path between two other nodes which helps to identify nodes that may be “hubs”); Closeness (average distance from the node to all other nodes in the network, i.e. a measure of how close a node is to all others nodes); and Strength (absolute sum of edge weights connected to node which helps to estimate the total involvement of a node in the network).

Statistical analyses were performed with SAS (release 9.4; SAS Statistical Institute, Cary, NC) and R Statistical Software version 4.0.3. All statistical tests were two-tailed. *p*-values were reported before and after adjustment for inclusion site (see Appendix for differences between sites). To minimize the risk of type 1 errors, we applied a Benjamini Hochberg correction for false discoveries (i.e., false discovery rate, FDR). *p*-values lower than 0.0005 were considered as significant [32].

## Results

### Sample description

We included 3,047 individuals with BD. Most individual were women (61.1%). The mean age at inclusion was 40.6 years old (±12.9). Regarding BD types, 45.6% of individuals were diagnosed with BD type I, 42.9% BD type II and 11.5% BD type NOS. The mean duration of illness was 17 years (±11.2). At inclusion, participants patients had a mean MADRS score of 10.3 (±9.0) and a mean YMRS score of 2.4 (±3.6).

**Figure 1.**
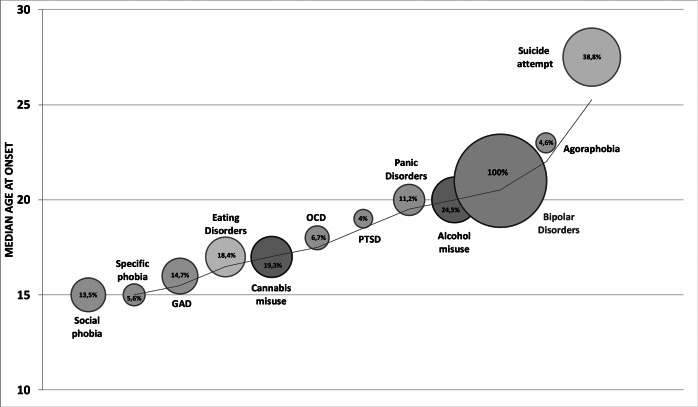
Diagrammatic representation of the prevalence and age at onset (AAO) of disorders. The location of the bubble on the vertical axis indicates the median AAO of each disorder. The size of each bubble is proportional to the prevalence of the disorder (e.g., the size of the bubble for bipolar disorders corresponds to 100%), with each percentage being indicated inside the bubble. Red: bipolar disorder; Blue: substance (alcohol and cannabis) use disorders; Green: anxiety disorders; Gray: eating disorders; and Orange: suicide attempts. GAD, generalized anxiety disorder; OCD, obsessive–compulsive disorder; PTSD, post-traumatic stress disorder.

Results about lifetime prevalence and AAO of psychiatric disorders and suicide attempts are presented in detail in Supplementary Table S1 and summarized in Figure 1. The six mostwere suicidal attempts (39%), alcohol misuse (25%), cannabis misuse (19%), eating disorders (18%), generalized anxiety disorder (15%), and social phobia (14%). The mean number of psychiatric comorbidities (excluding suicide attempt) per individual was 1.23 (SD = 1.4) (range 0–9, median = 1). Most comorbidities occurred between the ages of 15 and 20 years old and prior to the onset of BD. Panic disorders and alcohol misuse occurred the year before the onset of BD, while agoraphobia and suicidal attempts occurred after the onset of BD.

### Association between childhood maltreatment and psychiatric comorbidities

**Figure 2.**
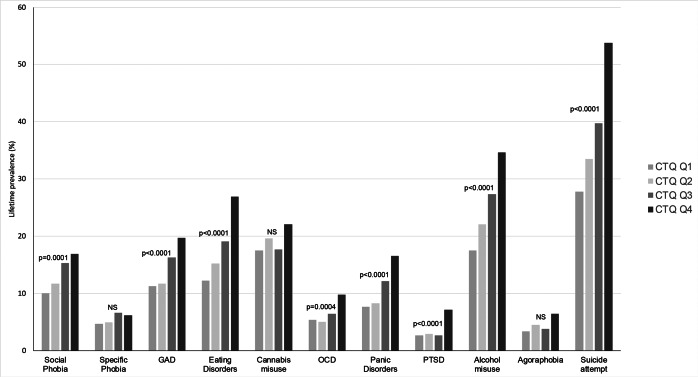
Lifetime prevalence of psychiatric comorbidities and suicide attempts according to childhood maltreatment severity (quartiles of CTQ total score). CTQ, childhood trauma questionnaire; GAD, generalized anxiety disorder; OCD, obsessive–compulsive disorder; PTSD, post-traumatic stress disorder; Q1–Q4, first, second, third, and fourth quartiles of CTQ total score. Psychiatric comorbidities and suicide attempt were ordered from left to right on the *X* axis by age at onset. *p*-values are given after adjutment for inclusion site.

With the exception of specific phobia, cannabis misuse and agoraphobia, most comorbidities were significantly more frequent in the CTQ upper quartile, this being highly significant for GAD, eating disorders, panic disorders, PTSD, alcohol misuse, and suicide attempts (*p* < 0.0001). In the CTQ upper quartile, the three most frequent comorbidities were suicide attempt (54%), alcohol use disorders (35%), and eating disorders (27%) (Figure 2 and details in Supplementary Table S2). We also observed that the mean number of comorbidities per individual was significantly higher in individuals exposed to higher levels of childhood maltreatment severity (*p* < 0.0001) (see Supplementary Table S4).

The age at onset of BD was earlier in individuals with a greater exposure to childhood maltreatment (*p* < 0.0001) (Supplementary Table S3). Regarding the sequence of onset of psychiatric comorbidities, there was no major difference in the median age at onset of comorbidities according to the quartiles of CTQ total score (data not shown in detail, described in Supplementary Table S3). There was a higher density of comorbidities (excluding suicide attempts) occurring prior to the onset of BD in individuals in the upper quartile of CTQ total score (*p* = 0.0004), which corresponds to a faster accumulation of psychiatric comorbidities prior to the onset of BD (see Supplementary Table S4).

**Figure 3.**
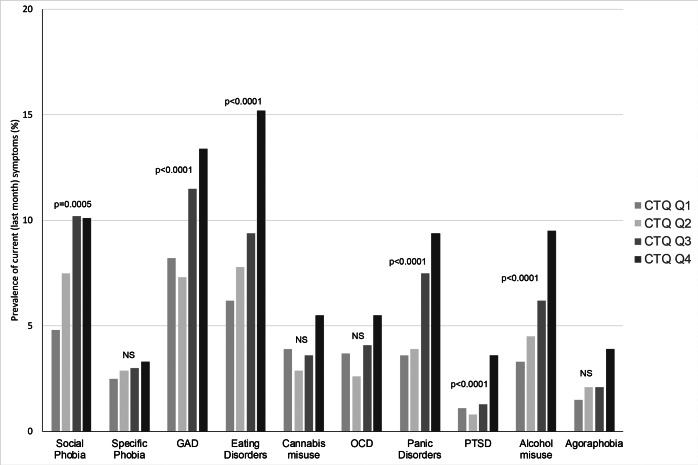
Prevalence of current symptoms (in the month before inclusion) for psychiatric comorbidities according to childhood maltreatment severity (quartiles of CTQ total score). CTQ, childhood trauma questionnaire; GAD, generalized anxiety disorder; OCD, obsessive–compulsive disorder; PTSD, post-traumatic stress disorder; Q1–Q4, first, second, third, and fourth quartiles of CTQ total score. Psychiatric comorbidities were ordered from left to right on the *X* axis by age at onset. *p*-values are given after adjutment for inclusion site.

The presence of symptoms during the last month before the assessment was more frequent for most comorbidities (with the exception of specific phobia, cannabis misuse, OCD, and agoraphobia) in the CTQ total score upper quartile (Figure 3 and details in Supplementary Table S5). These differences were highly significant for GAD, eating disorders, panic disorders, PTSD, and alcohol misuse, which were all more active during the last month before inclusion in the CTQ total score upper quartile (all *p* < 0.0001).

### Association between psychiatric comorbidities and CTQ subtypes

**Figure 4.**
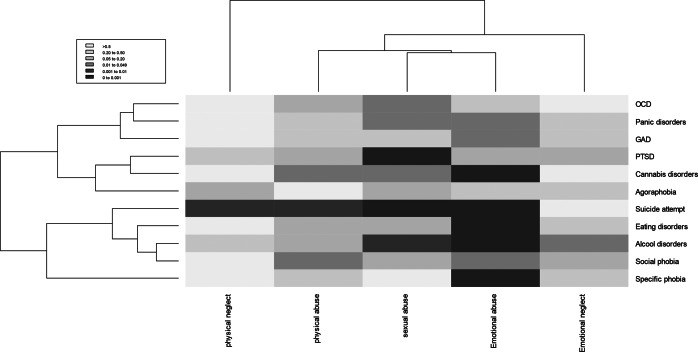
Association between psychiatric comorbidities, suicide attempts, and subscores of childhood trauma questionnaire (heatmap based on *p*-values). GAD, generalized anxiety disorder; OCD, obsessive–compulsive disorder; PTSD; post-traumatic stress disorder. *p*-values were adjusted for age, sex, BD type, and MADRS scores.

We tested the associations between the lifetime prevalence of each comorbidity and the five CTQ subscores in a multivariable logistic regression analysis, with an adjustment for age, sex, BD type, and MADRS scores. Data were not shown in details, but summarized in an heatmap (Figure 4).

Light and dark red rectangles in the heatmap corresponded to associations with *p*-values < 0.001. Emotional abuse was the only subtype of maltreatment that was associated with a wide range of different comorbidities, mainly cannabis and alcohol use disorders, eating disorders, and specific phobia, but also with suicide attempt. In turn, suicide attempt was associated with a wide range of maltreatment (emotional abuse, sexual abuse, physical abuse, and physical neglect). Both emotional abuse and sexual abuse were associated with alcohol use disorders. PTSD was associated only with sexual abuse.

### Network analysis of childhood abuse, psychiatric comorbidities, and suicide attempts

Since maltreatment subtypes may coexist, which is also the case for psychiatric comorbidities and suicide attempts, we used a network analysis to visualize the links between maltreatment subtypes, comorbidities, and suicide attempts. Since neglect was associated only with suicide attempts, it was not included in the analysis which was therefore performed only with the three subtypes of abuse (emotional, physical, and sexual abuse). For a question of clarity, panic disorders and agoraphobia were pooled into a single category.

**Figure 5.**
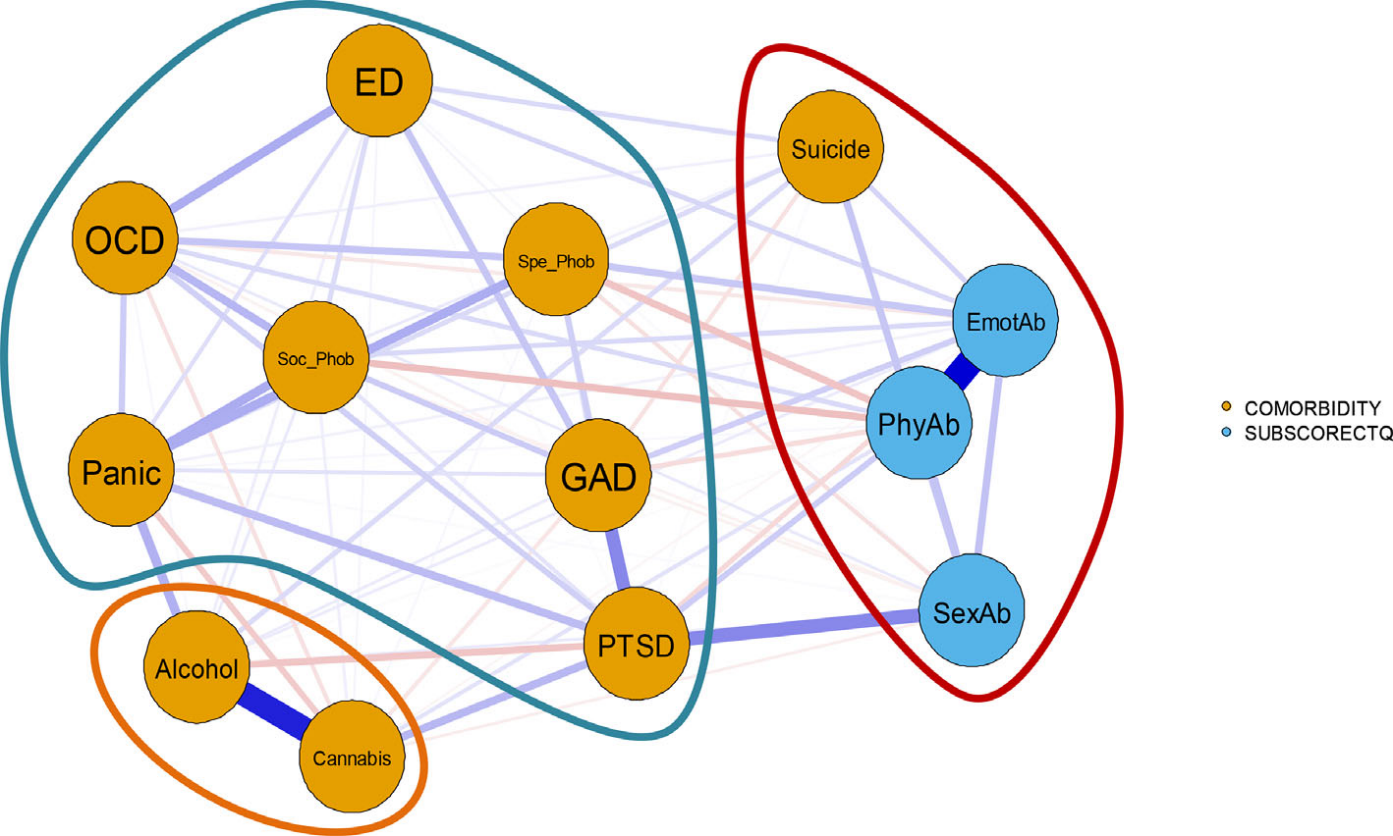
Network plot of CTQ abuse subtypes, psychiatric comorbidities, and suicide attempts. Orange circles correspond to psychiatric comorbidities and suicide attempts, while blue circles correspond to subscores of the CTQ for sexual abuse (SexAb), physical abuse (PhyAb), and emotional abuse (EmotAb). Community 1 (in red) included suicide attempts and all abuses. Community 2 (in orange) included alcohol and cannabis use disorders. Community 3 (in blue) included eating disorders (ED) and anxiety disorders (GAD, generalized anxiety disorder; OCD, obsessive–compulsive disorder; PTSD, post-traumatic stress disorder, panic disorder [including agoraphobia]; Soc-Phob, social phobia; Spe-Phob, specific phobia).

The graph (see Figure 5) identified a first community (in red) which included the three forms of abuse and suicide attempts, a second community (in orange) which included only alcohol and cannabis use disorders, and a third community (in blue) which included all anxiety disorders and eating disorders. There were few direct edges between communities 1 and 2. PTSD had higher indices of strength, closeness, and betweenness, suggesting that PTSD is more central in the network. Emotional abuse had also a high index of strength, meaning that it is highly connected to the other variables within the network. Centrality indices are shown in Supplementary Figure S1. Bootstrapping indicates high stability of edge and centrality indices for this sample (Supplementary Figure S2).

## Discussion

This large sample of individuals with BD offers the opportunity to disentangle the associations between lifetime psychiatric comorbidities, suicide attempts, and childhood maltreatment. Most psychiatric comorbidities were more frequent, but also more persistent in individuals having been exposed to more severe childhood maltreatment (i.e., CTQ total score in the upper quartile). Childhood maltreatment did not modify the chronology of onset of comorbidities. However, individuals with higher levels of childhood maltreatment had a faster accumulation of multiple comorbidities prior to the onset of BD as compared to those not exposed. When disentangling links between maltreatment subtypes, psychiatric comorbidities, and suicide attempts, we observed that most were associated with emotional abuse, but also with sexual and/or physical abuse (in the case of suicide attempts or alcohol use disorders). The network analysis identified strong links between all types of abuse and suicide attempts, but also links with anxiety disorders.

This study reinforces findings from previous meta-analyses about the high lifetime prevalence of psychiatric comorbidities and suicide attempts in BD [3–5, 7–9, 33]. The five most prevalent comorbidities in this sample were AUD, CUD, eating disorders, GAD, and social phobia. Lifetime prevalence of suicide attempts was also very high in this sample (39%) and was close to the ones reported in previous meta-analyses (32–36%) [9,10]. Apart from suicide attempts and agoraphobia, most comorbidities occurred prior to the onset of BD. This observation is not fully consistent with our previously published article (using data obtained in a smaller sample and as part of a research protocol in genetics) which suggested that panic disorder, agoraphobia, alcohol misuse, and suicide attempts mainly occurred after the onset of BD [12]. While rates of lifetime comorbidities are consistently reported as high in BD, further studies are therefore required to clarify their chronology of onset.

This study not only replicates findings from the literature demonstrating that childhood maltreatment is associated with higher lifetime prevalence of most psychiatric comorbidities and suicide attempts [20], but adds several new findings. First, childhood maltreatment was associated not only with the lifetime presence of comorbidities but also with the persistence or chronicity of clinical symptoms for most of them. Indeed, symptoms during the last month before assessment were more frequent for most comorbidities in the CTQ total score upper quartile as compared to those individuals with lower levels (or absence) of maltreatment. In the CTQ total score upper quartile, current symptoms of eating disorder, GAD, social phobia, and alcohol use disorders were frequent. Such results are consistent with those obtained in the NESDA study (Netherlands Study of Depression and Anxiety) suggesting that childhood maltreatment was associated with persistence and chronicity of comorbidities in adults with anxiety disorders [34–36]. This implies that childhood maltreatment may render comorbidities more chronic, more complex to treat, and/or less responsive to interventions. As such, childhood maltreatment should be systematically screened in any individual with psychiatric comorbidities, especially when these comorbidities have a chronic or persistent course.

Second, this study provides fine-grained association analyses between psychiatric comorbidities, suicide attempts, and maltreatment subtypes. Among maltreatment subtypes, emotional abuse was the main associated factor (for cannabis misuse, eating disorders, and specific phobia), sometimes in combination with sexual abuse (for alcohol use disorders) or with a broader range of maltreatment subtypes (for suicide attempts). Hence, this suggests that emotional abuse may play a central role in the risk of developing psychiatric comorbidities in BD. Childhood maltreatment has also been associated with the prevalence of suicide attempts in at least two independent meta-analyses [20,37]. Noteworthy, effect sizes for the associations with suicide attempts were the highest for both sexual abuse and emotional abuse and the lowest for physical neglect [37]. In this sample, we observed very similar findings, suggesting that the main determinants of suicide attempts might be emotional abuse and sexual abuse. These results may be important for future research that would aim at examining the outcomes in BD, but should avoid aggregating all maltreatment subtypes into a single category.

Finally, it has been demonstrated that childhood maltreatment decreases the age at onset of BD [17, 20, 38], which is also observed in this study. We hypothesized that childhood maltreatment might also decrease the age at onset for comorbidities. For example, some studies from the NESARC samples (National Epidemiological Survey on Alcohol and Related Conditions) suggested that childhood maltreatment might increase the speed of transition from first alcohol use, to regular drinking and alcohol use disorder [39], in particular in women [40] and might also lead to a faster transition from cannabis use to CUD in individuals exposed to more childhood adversities [41]. Regarding anxiety disorders, data from the literature are less consistent [42], but some suggested that anxiety disorders may occur earlier in individuals with childhood adversities [43]. Our hypothesis that childhood maltreatment would decrease the age at onset for comorbidities is not supported by our study. Nevertheless, we suggest a faster accumulation of multiple comorbidities prior to the onset of BD in individuals with a higher exposure to childhood maltreatment. As a whole, in this sample, and regardless of childhood maltreatment, some psychiatric comorbidities (GAD, CUD, AUD, PTSD, and panic disorders) seemed to occur earlier as compared to what would be expected in the general population, this being described in a meta-analysis of 192 epidemiological studies [44].

This study has several strengths, including a comprehensive clinical assessment of psychiatric comorbidities and suicide attempts and a large sample size. Nevertheless, several limitations deserve some comments. Lifetime prevalence, age at onset of disorders and childhood maltreatment, have been assessed retrospectively, which is therefore potentially hampered by memory biases, and tendencies for over or under estimation. The use of the CTQ to assess childhood maltreatment has been questioned in the literature, but appears robust in clinical samples. For example, when comparing the CTQ and a comprehensive interview for childhood experiences of care and abuse in cases with first-episode psychosis and controls, it has been reported fair levels of agreement and reasonably high convergent validity for reports of sexual and emotional abuse, while convergent validity for physical abuse was slightly lower in cases as compared with controls [45]. Minimization and denial may also be common when using self-reports of childhood trauma [29], however, some results indicated that minimization-denial may be—in fact—more frequent in controls as compared to cases with severe psychiatric disorders (i.e., schizophrenia and mood disorders) [46]. Moreover, the CTQ does not provide precise information about some characteristics of trauma that may be important to consider (onset of exposure, cumulative duration of exposure, etc.) and that would require further investigations. Although used in some previous studies [29, 30], the use of the CTQ total score divided in quartiles may be questioned. To date, there is no consensus about the most appropriate way to identify individuals who were exposed to severe/multiple forms of childhood maltreatment. The CTQ total score can be used as a continuous variable (eventually divided in quartiles of severity), while severe/multiple maltreatment can be defined by an ordinal variable (number of subtypes of trauma of at least a moderate severity) or by a binary variable (childhood maltreatment being considered present if any type of abuse or neglect was rated as moderate or severe) [47,48]. Finally, the representativeness of the sample used in this study may be questioned since individuals with very high or very low rates of psychiatric comorbidities might have been less likely to be referred to the network, because not enough stabilized (for the first ones) or not requiring any advice from tertiary universities-affiliated services (for the second ones).

In conclusion, we found significant associations between childhood maltreatment and the prevalence and persistence of numerous psychiatric comorbidities, but also associations with the suicidal risk in BD. Psychiatric comorbidities accumulated faster prior to the onset of BD in individuals exposed to more severe/multiple childhood trauma. Among maltreatment subtypes, emotional abuse and sexual abuse may have a central role in this comorbid clinical profile. Hence, childhood maltreatment should be systematically assessed in individuals with BD, in particular when the course of BD is characterized by multiple and/or persistent psychiatric comorbidities or by suicide attempts. Since both childhood maltreatment and psychiatric comorbidities may increase the risk of subsequent relapses, but also may condition trajectories of poor functioning [24,49] in BD, they are key components that should be systematically screened during the clinical assessment to better prescribe personalized therapeutic strategies and psychosocial interventions.

## Data Availability

The data that support the findings of this study are available on request from the corresponding author. The data are not publicly available because of privacy or ethical restrictions.

## Supplementary Materials

To view supplementary material for this article, please visit http://doi.org/10.1192/j.eurpsy.2022.7. click here to view supplementary material

## Appendix

List of FondaMental Advanced Center of Expertise for Bipolar Disorders (FACE-BD) Collaborators:

FACE-BD Clinical Coordinating Center (Fondation FondaMental): B. Etain, E. Olié, M. Leboyer, and P. M. Llorca; FACE-BD Data Coordinating Center (Fondation FondaMental): V. Barteau, S. Bensalem, O. Godin, H. Laouamri, and K. Souryis; FACE-BD Clinical Sites and Principal Collaborators in France: AP-HP, DHU PePSY, Pôle de Psychiatrie et d’Addictologie des Hôpitaux Universitaires H Mondor, Créteil, France—S. Hotier, A. Pelletier, J. P. Sanchez, E. Saliou, C. Hebbache, J. Petrucci, L. Willaume, and E. Bourdin; APHP, Groupe Hospitalo-Universitaire AP-HP Nord, DMU Neurosciences, Département de Psychiatrie et Médecine Addictologie, Hôpital Fernand Widal, Paris, France—F. Bellivier, M. Carminati, B. Etain, E. Marlinge, J. Meheust, and C. Zekri-Lachgar; Hôpital C. Perrens, Centre Expert Trouble Bipolaire, Service de Psychiatrie Adulte, Pôle 3-4-7, Bordeaux, France—B. Antoniol, A. Desage, S. Gard, A. Jutant, K. Mbailara, I. Minois, and L. Zanouy; Département d’Urgence et Post Urgence Psychiatrique, CHRU Montpellier, Montpellier, France—L. Bardin, P. Courtet, B. Deffinis, D. Ducasse, M. Gachet, F. Molière, B. Noisette, E. Olié, and G. Tarquini; Pôle de Psychiatrie, Addictologie et Pédopsychiatrie, Hôpital Sainte Marguerite, Marseille, France—R. Belzeaux, J. L. Consoloni, F. Groppi, E. Moreau, A. Lefrere, L. Lescalier, and N. Viglianese; Université de Lorraine, Centre Psychothérapique de Nancy, Inserm U1114, Nancy, France—R. Cohen, G. Gross, R. Schwan, T. Schwitzer, and O. Wajsbrot-Elgrabli; Service Universitaire de Psychiatrie, CHU de Grenoble et des Alpes, Grenoble, France—T. Bougerol, B. Fredembach, A. Suisse, A Pouchon, and M. Polosan; Centre Hospitalier de Versailles, Service Universitaire de Psychiatrie d’Adultes et d’Addictologie, Le Chesnay, France—A. S. Cannavo, A. Créa, V. Feuga, A. M. Galliot, N. Kayser, C. Passerieux, and P. Roux; Service de Psychiatrie, Centre Hospitalier Princesse Grace, Monaco—V. Aubin, I. Cussac, M. A. Dupont, J. Loftus, I. Medecin, and T. Burté; APHP, Groupe Hospitalo-Universitaire AP-HP Nord, DMU ESPRIT, Service de Psychiatrie et Addictologie, Hôpital Louis Mourier, Colombes, France—C. Dubertret, N. Mazer, C. Portalier, C. Scognamiglio, and A. Bing; Service de Psychiatrie de l’Adulte, CHU de Besançon—C. Aymé, D. Bennabi, E. Haffen, and E. Mercelat.

**Table A1.** Sociodemographical and clinical characteristics by site of recruitment.

	Sites of recruitment												
Variables *n* (%) or mean (SD)	Bordeaux	Créteil	Montpellier	Grenoble	Nancy	Marseille	Paris	Versailles	Monaco	Clermont	Colombes	Besançon	*p* value
Sex (female)	60.5%	63.7%	65.9%	61.1%	61.4%	64.1%	58.0%	57.4%	58.3%	51.2%	51.7%	51.2%	0.10
Age at inclusion	38.0 (13.2)	39.4 (11.9)	40.5 (12.9)	43.3 (13.3)	44.1 (13.4)	39.2 (12.5)	39.7 (12.4)	39.9 (12.3)	34.1 (11.7)	44.7 (12.5)	39.8 (12.8)	49.8 (11.8)	<0.0001
BD subtype													
BD I	64.4%	48.3%	47.0%	46.9%	42.2%	32.6%	58.8%	36.5%	51.2%	29.3%	36.2%	46.4%	<0.0001
BD II	31.0%	46.2%	39.0%	41.6%	38.6%	50.4%	34.4%	53.4%	34.5%	68.3%	55.2%	46.3%	
BD NOS	4.6%	5.5%	14.0%	11.5%	19.2%	17.0%	6.8%	10.1%	14.3%	2.4%	8.6%	7.3%	
Duration of BD	14.7 (10.5)	16.0 (10.2)	17.3 (11.2)	17.7 (12.0)	18.4 (11.8)	17.5 (11.2)	15.9 (11.1)	16.6 (10.6)	14.8 (10.5)	19.0 (11.8)	15.2 (9.8)	24.7 (11.5)	<0.0001
MADRS score	8.4 (8.2)	11.1 (9.3)	10.8 (9.5)	10.3 (8.9)	11.3 (10.0)	10.2 (8.7)	8.3 (7.9)	10.9 (8.6)	14.2 (11.4)	12.9 (8.7)	7.3 (6.5)	10.7 (8.3)	<0.0001
YMRS score	1.8 (2.9)	2.5 (3.6)	2.6 (4.1)	2.7 (3.7)	2.2 (3.2)	2.8 (4.0)	1.5 (2.8)	2.3 (3.4)	3.9 (4.4)	4.0 (4.8)	2.0 (3.8)	2.2 (2.3)	<0.0001
Age at onset of BD	23.1 (8.9)	23.4 (9.0)	23.1 (9.7)	25.3 (10.6)	25.8 (10.5)	22.1 (7.8)	23.5 (8.3)	23.4 (8.9)	19.7 (6.8)	25.6 (9.7)	24.0 (9.0)	25.0 (8.8)	<0.0001
CTQ total score	41.7 (14.5)	40.9 (12.8)	44.4 (15.9)	40.4 (13.4)	42.5 (14.6)	44.8 (15.8)	41.1 (12.9)	43.4 (14.5)	43.2 (15.3)	44.2 (14.9)	43.2 (14.9)	47.6 (19.8)	<0.0001
Social phobia	14 (10.8%)	70 (24.0%)	103 (20.1%)	23 (6.2%)	37 (12.0%)	45 (11.5%)	57 (12.9%)	31 (8.3%)	22 (26.2%)	6 (14.6%)	1 (1.7%)	2 (4.9%)	<0.0001
Specific phobia	3 (2.3%)	28 (9.6%)	25 (4.9%)	12 (3.2%)	9 (2.9%)	30 (7.6%)	24 (5.4%)	21 (5.6%)	14 (16.7%)	0	4 (6.9%)	0	<0.0001
GAD	8 (6.2%)	68 (23.3%)	124 (24.2%)	58 (15.6%)	32 (10.4%)	62 (15.8%)	45 (10.2%)	26 (7.0%)	11 (13.1%)	3 (7.3%)	5 (8.6%)	7 (17.1%)	<0.0001
Eating disorders	20 (15.5%)	58 (19.9%)	93 (18.1%)	64 (17.2%)	78 (25.3%)	102 (25.9%)	48 (10.9%)	61 (16.4%)	25 (29.8%)	1 (2.4%)	9 (15.5%)	3 (7.3%)	<0.0001
Cannabis misuse	18 (14.0%)	55 (18.8%)	111 (21.6%)	67 (18.0%)	43 (14.0%)	88 (22.4%)	86 (19.5%)	58 (15.6%)	25 (29.8%)	8 (19.5%)	25 (43.1%)	4 (9.8%)	<0.0001
OCD	2 (1.6%)	18 (6.2%)	43 (8.4%)	16 (4.3%)	38 (12.3%)	31 (7.9%)	27 (6.1%)	18 (4.8%)	4 (4.8%)	3 (7.3%)	1 (1.7%)	2 (4.9%)	0.0004
Panic disorders	10 (7.7%)	40 (13.7%)	55 (10.7%)	43 (11.5%)	49 (15.9%)	46 (11.7%)	32 (7.2%)	57 (15.3%)	2 (2.4%)	2 (4.9%)	3 (5.2%)	0	0.0002
PTSD	4 (3.1%)	31 (10.6%)	18 (3.5%)	13 (3.5%)	8 (2.6%)	14 (3.6%)	24 (5.4%)	4 (1.1%)	3 (3.6%)	0	0	10 (24.4%)	<0.0001
Alcohol misuse	21 (16.3%)	79 (27.1%)	148 (28.8%)	83 (22.3%)	84 (27.3%)	102 (25.9%)	98 (22.2%)	85 (22.8%)	26 (30.9%)	14 (34.2%)	25 (43.1%)	0	0.003
Agoraphobia	6 (4.6%)	11 (3.8%)	41 (8.0%)	14 (3.8%)	20 (6.5%)	20 (5.1%)	12 (2.7%)	9 (2.4%)	5 (5.9%)	0	1 (1.7%)	19 (46.3%)	0.001
Suicide attempt	47 (36.4%)	104 (35.6%)	234 (45.6%)	139 (37.3%)	126 (40.9%)	159 (40.5%)	142 (32.1%)	139 (37.3%)	36 (42.9%)	18 (43.9%)	20 (34.5%)	19 (46.3%)	0.012

Abbreviations: BD, bipolar disorder; CTQ, childhood trauma questionnaire; GAD, generalized anxiety disorder; MADRS, Montgomery Asberg depression rating scale; OCD, obsessive–compulsive disorder; PTSD, post-traumatic stress disorder; SD, standard deviation; YMRS, Young Mania rating scale.
