# Supplementary material for: Influence of childhood maltreatment on prevalence, onset, and persistence of psychiatric comorbidities and suicide attempts in bipolar disorders
Source: Eur Psychiatry. 2022 Jan 22;65(1):e15. doi: 10.1192/j.eurpsy.2022.7 (PMC8853858; doi:10.1192/j.eurpsy.2022.7)

**Supplementary Table S1:**

**Prevalence and age at onset (AAO) of psychiatric comorbidities (ordered from lowest to highest median AAO)**

|  | N present | Cumulative prevalence (%) | AAO (median) | IQR for AAO |
| --- | --- | --- | --- | --- |
| Social Phobia | 411 | 13.5% | 15 | 10-19 |
| Specific Phobia | 170 | 5.6% | 15 | 10-22 |
| GAD | 449 | 14.7% | 16 | 12-21 |
| Eating Disorders | 562 | 18.4% | 17 | 14-23 |
| Cannabis misuse | 588 | 19.3% | 17 | 15-20 |
| OCD | 203 | 6.7% | 18 | 13-25 |
| PTSD | 119 | 4.0% | 19 | 14-28 |
| Panic Disorders | 341 | 11.2% | 20 | 15-30 |
| Alcohol misuse | 775 | 25.4% | 20 | 17-30 |
| **Median AAO of bipolar disorders= 21 years (17-28)** | | | | |
| Agoraphobia | 139 | 4.6% | 23 | 17-32 |
| Suicide attempt | 1183 | 38.8% | 27.5 | 19-39 |

*AAO: Age at Onset, IQR: InterQuartile, OCD: Obsessive-Compulsive disorder, PTSD: Post-Traumatic stress disorder.*

**Supplementary** **Table S2:**

**Lifetime prevalence of psychiatric comorbidities and suicide attempts (N and %) according to childhood maltreatment severity (quartiles of CTQ total score)**

|  | **Childhood maltreatment severity** | | | | **P values** | **P values adjusted for inclusion site** |
| --- | --- | --- | --- | --- | --- | --- |
| **Quartiles of CTQ total score (n)** | **Q1 (n=727)** | **Q2 (n=815)** | **Q3 (n=718)** | **Q4 (n=787)** |  |  |
| **Range of CTQ total score** | **25-32** | **32 – 40** | **40 - 50** | **50-125** |  |  |
| Social Phobia | 73 (10.0) | 95 (11.7) | 110 (15.3) | 133 (16.9) | **0.0002** | **0.0001** |
| Specific Phobia | 34 (4.7) | 40 (4.9) | 47 (6.6) | 49 (6.2) | 0.29 | 0.25 |
| GAD | 82 (11.3) | 95 (11.7) | 117 (16.3) | 155 (19.7) | **<0.0001** | **<0.0001** |
| Eating Disorders | 89 (12.2) | 124 (15.2) | 137 (19.1) | 212 (26.9) | **<0.0001** | **<0.0001** |
| Cannabis misuse | 127 (17.5) | 160 (19.6) | 127 (17.7) | 174 (22.1) | 0.08 | 0.11 |
| OCD | 39 (5.4) | 41 (5.0) | 46 (6.4) | 77 (9.8) | **0.0005** | **0.0004** |
| Panic Disorders | 56 (7.7) | 68 (8.3) | 87 (12.1) | 130 (16.5) | **<0.0001** | **<0.0001** |
| PTSD | 20 (2.7) | 24 (2.9) | 19 (2.7) | 56 (7.1) | **<0.0001** | **<0.0001** |
| Alcohol misuse | 127 (17.5) | 180 (22.1) | 196 (27.3) | 272 (34.6) | **<0.0001** | **<0.0001** |
| Agoraphobia | 25 (3.4) | 37 (4.5) | 27 (3.8) | 50 (6.4) | 0.03 | 0.04 |
| Suicide attempt | 202 (27.8) | 273 (33.5) | 285 (39.7) | 423 (53.8) | **<0.0001** | **<0.0001** |

*CTQ: Childhood Trauma Questionnaire, OCD: Obsessive Compulsive Disorder, GAD: Generalized Anxiety Disorder, PTSD: Post-Traumatic Stress Disorder.*

*In bold, p values considered as significant (p<0.0005)*

**Supplementary Table S3:**

**Median age at onset (and IQR) of psychiatric comorbidities and suicide attempts according to childhood maltreatment severity (quartiles of CTQ total score)**

|  | **Childhood maltreatment severity** | | | | **P value** | **P values adjusted for inclusion site** |
| --- | --- | --- | --- | --- | --- | --- |
| **Quartiles of CTQ total score (n)** | **Q1 (n=727)** | **Q2 (n=815)** | **Q3 (n=718)** | **Q4 (n=787)** |  |  |
| **Range of CTQ total score** | **25-32** | **32 – 40** | **40 - 50** | **50-125** |  |  |
| Social Phobia | 15 (10-17) | 14 (10-17) | 15 (12-20) | 14 (10-18) | 0.30 | 0.04 |
| Specific Phobia | 12 (7-15) | 15 (13-23) | 17.5 (11-22) | 13 (10-25) | 0.06 | 0.06 |
| GAD | 17 (11-22) | 16 (13-20) | 17 (12-21) | 16 (11-25) | 0.97 | 0.74 |
| Eating Disorders | 17 (15-22) | 18 (15-22) | 17 (14-25) | 16 (14-23) | 0.73 | 0.56 |
| Cannabis misuse | 17 (15-19) | 17 (15-20) | 18 (16-20) | 17 (15-20) | 0.17 | 0.19 |
| OCD | 16 (11-20) | 18 (10-22) | 21.5 (16-30) | 16 (13-23) | 0.02 | 0.04 |
| Panic Disorders | 18 (15-21) | 20 (15-34) | 21 (15-32) | 21 (15-30) | 0.36 | 0.29 |
| PTSD | 21.5 (18-30) | 24.5 (16-37) | 18 (12-24) | 17 (10-23) | 0.01 | 0.06 |
| Alcohol misuse | 20 (17-30) | 20 (16-28) | 20 (17-30) | 21 (16-30) | 0.68 | 0.41 |
| Agoraphobia | 18 (14-37) | 25 (19-40) | 26.5 (18-31) | 20 (17-32) | 0.63 | 0.77 |
| Suicide attempt | 30 (21-42) | 25.4 (19-37) | 27.5 (20-37) | 28 (18-40) | 0.04 | 0.05 |
| Bipolar Disorder | 22 (18-28) | 21 (17-28) | 21 (17-28) | 20 (16-26) | **p<0.0001** | **p<0.0001** |

*CTQ: Childhood Trauma Questionnaire, OCD: Obsessive Compulsive Disorder, GAD: Generalized Anxiety Disorder, PTSD: Post-Traumatic Stress Disorder.*

*In bold, p values considered as significant (p<0.0005)*

**Supplementary Table S4:**

**Mean number and density of comorbidities (excluding suicidal attempt) before and after the onset of BD according to childhood maltreatment severity (quartiles of CTQ total score)**

|  | **Childhood maltreatment severity** | | | | **P values** | **P values adjusted for inclusion site** |
| --- | --- | --- | --- | --- | --- | --- |
| **Quartiles of CTQ total score (n)** | **Q1 (n=727)** | **Q2 (n=815)** | **Q3 (n=718)** | **Q4 (n=787)** |  |  |
| **Range of CTQ total score** | **25-32** | **32 – 40** | **40 - 50** | **50-125** |  |  |
| Mean number of comorbidities per individual | 0.9 (1.2) | 1.1 (1.2) | 1.3 (1.4) | 1.7 (1.6) | **<0.0001** | **<0.0001** |
| Density of comorbidities  before the onset of BD | 0.049 (0.06) | 0.051 (0.06) | 0.053 (0.07) | 0.065 (0.07) | **0.0005** | **0.0004** |
| Density of comorbidities  after the onset of BD | 0.048 (0.10) | 0.056 (0.14) | 0.057 (0.10) | 0.046 (0.07) | 0.41 | 0.61 |

*Density before the onset of BD: number of comorbidities occurring before the onset of BD divided by the age at onset of BD*

*Density after the onset of BD: number of comorbidities occurring after the onset of BD divided by the duration of BD*

*In bold, p values considered as significant (p<0.0005)*

**Supplementary Table S5:**

**Prevalence of current symptoms (in the month before inclusion) for psychiatric comorbidities (N and %) according to childhood maltreatment severity (quartiles of CTQ total score)**

|  | **Childhood maltreatment severity** | | | | **P values** | **P values adjusted for inclusion site** |
| --- | --- | --- | --- | --- | --- | --- |
| **Quartiles of CTQ total score (n)** | **Q1 (n=727)** | **Q2 (n=815)** | **Q3 (n=718)** | **Q4 (n=787)** |  |  |
| **Range of CTQ total score** | **25-32** | **32 – 40** | **40 - 50** | **50-125** |  |  |
| Social Phobia | 34 (4.8) | 60 (7.5) | 71 (10.2) | 75 (10.1) | **0.0002** | **0.0005** |
| Specific Phobia | 18 (2.5) | 23 (2.9) | 21 (3.0) | 25 (3.3) | 0.86 | 0.89 |
| GAD | 58 (8.2) | 57 (7.3) | 79 (11.5) | 99 (13.4) | **0.0001** | **<0.0001** |
| Eating Disorders | 45 (6.2) | 63 (7.8) | 67 (9.4) | 117 (15.2) | **<0.0001** | **<0.0001** |
| Cannabis misuse | 27 (3.9) | 22 (2.9) | 24 (3.6) | 40 (5.5) | 0.06 | 0.07 |
| OCD | 27 (3.7) | 21 (2.6) | 29 (4.1) | 42 (5.5) | 0.03 | 0.03 |
| Panic Disorders | 25 (3.6) | 30 (3.9) | 51 (7.5) | 68 (9.4) | **<0.0001** | **<0.0001** |
| PTSD | 8 (1.1) | 6 (0.8) | 9 (1.3) | 28 (3.6) | **<0.0001** | **<0.0001** |
| Alcohol misuse | 23 (3.3) | 34 (4.5) | 41 (6.2) | 68 (9.5) | **<0.0001** | **<0.0001** |
| Agoraphobia | 11 (1.5) | 17 (2.1) | 17 (2.1) | 30 (3.9) | 0.02 | 0.03 |

*CTQ: Childhood Trauma Questionnaire, OCD: Obsessive Compulsive Disorder, GAD: Generalized Anxiety Disorder, PTSD: Post-Traumatic Stress Disorder.*

*In bold, p values considered as significant (p<0.0005)*

**Supplementary figure 1: Indices of centrality of the network**

**Supplementary figure 2: stability coefficient of strength**


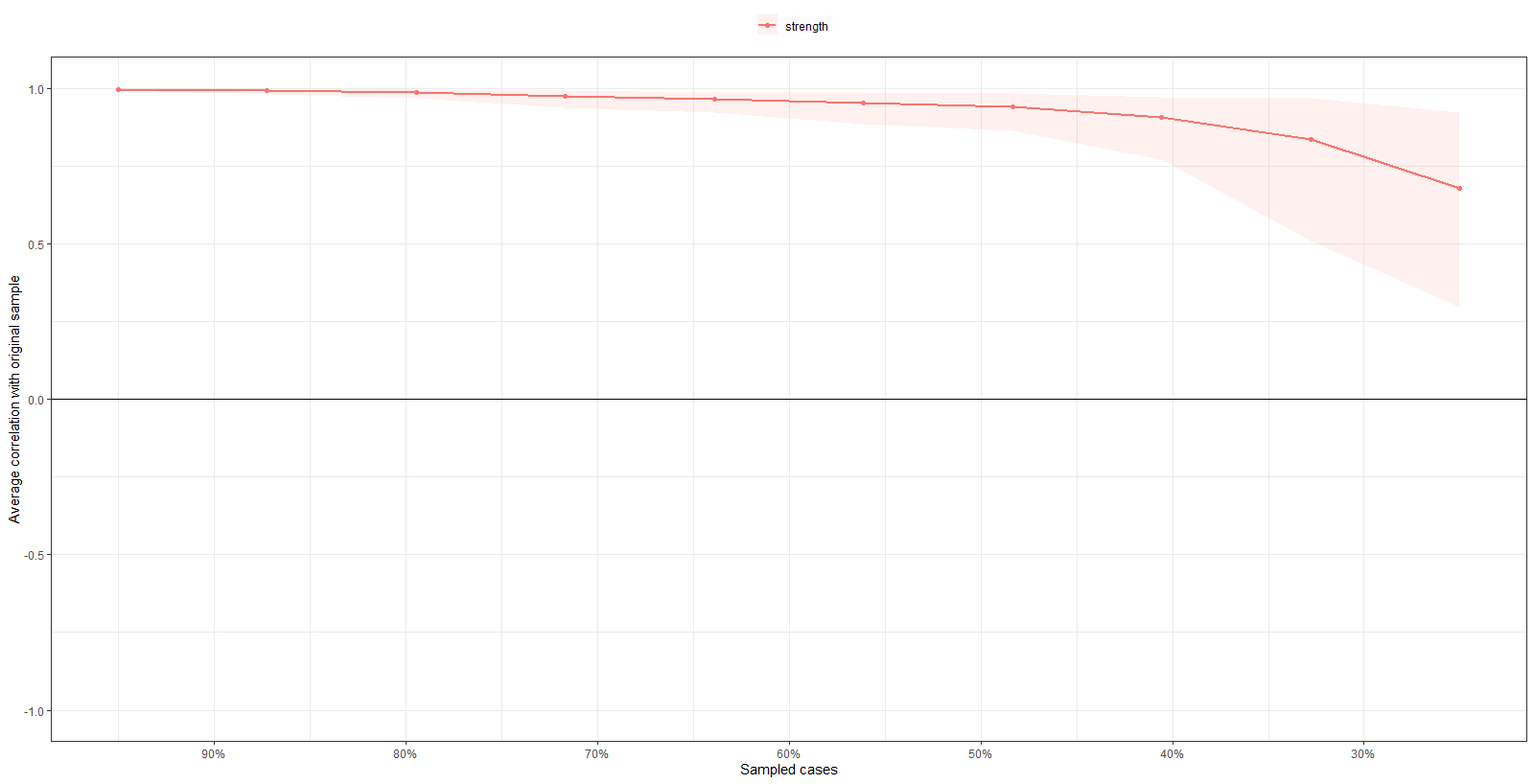

Supplement: Supplementary file 1 [file S0924933822000074sup001.docx]
